# A consensus linkage map of the grass carp (*Ctenopharyngodon idella*) based on microsatellites and SNPs

**DOI:** 10.1186/1471-2164-11-135

**Published:** 2010-02-24

**Authors:** Jun Hong Xia, Feng Liu, Ze Yuan Zhu, Jianjun Fu, Jianbin Feng, Jiale Li, Gen Hua Yue

**Affiliations:** 1 Molecular Population Genetics Group, Temasek Life Sciences Laboratory, National University of Singapore, 117604 Republic of Singapore; 2 Key Laboratory of Exploration and Utilization of Aquatic Genetic Resources, Shanghai Ocean University, Ministry of Education, Shanghai 201306, China; 3 Aquaculture Division, E-Institute of Shanghai Universities, Shanghai 201306, China

## Abstract

**Background:**

Grass carp (*Ctenopharyngodon idella*) belongs to the family Cyprinidae which includes more than 2000 fish species. It is one of the most important freshwater food fish species in world aquaculture. A linkage map is an essential framework for mapping traits of interest and is often the first step towards understanding genome evolution. The aim of this study is to construct a first generation genetic map of grass carp using microsatellites and SNPs to generate a new resource for mapping QTL for economically important traits and to conduct a comparative mapping analysis to shed new insights into the evolution of fish genomes.

**Results:**

We constructed a first generation linkage map of grass carp with a mapping panel containing two F1 families including 192 progenies. Sixteen SNPs in genes and 263 microsatellite markers were mapped to twenty-four linkage groups (LGs). The number of LGs was corresponding to the haploid chromosome number of grass carp. The sex-specific map was 1149.4 and 888.8 cM long in females and males respectively whereas the sex-averaged map spanned 1176.1 cM. The average resolution of the map was 4.2 cM/locus. BLAST searches of sequences of mapped markers of grass carp against the whole genome sequence of zebrafish revealed substantial macrosynteny relationship and extensive colinearity of markers between grass carp and zebrafish.

**Conclusions:**

The linkage map of grass carp presented here is the first linkage map of a food fish species based on co-dominant markers in the family Cyprinidae. This map provides a valuable resource for mapping phenotypic variations and serves as a reference to approach comparative genomics and understand the evolution of fish genomes and could be complementary to grass carp genome sequencing project.

## Background

A linkage map is an essential framework for mapping traits of interest and is often the first step towards understanding genome evolution and aids genome assembly [1-4]. With the advent of molecular genetic techniques and sophisticated statistical tools for linkage analysis, linkage maps have been constructed for some economically important aquaculture species, such as channel catfish, tilapia, Asian seabass, European sea bass, shrimps, and oysters [4]. Until last decade, dominant DNA markers such as AFLP and RAPD were used to construct linkage maps that are now replaced by co-dominant markers such as microsatellites and SNPs. Microsatellites have been extensively used in construction of genetic maps for some aquaculture fishes recently, e.g., brown trout, *Salmo trutta *[5], nile tilapia, *Oreochromis niloticus *[6], channel catfish, *Ictalurus punctatus *[7,8], Asian seabass, *Lates calcarifer *[9] and blacklip abalone, *Haliotis rubra *[10]. With the rapid development of DNA sequencing and genotyping technologies, single nucleotide polymorphism (SNP) [11], the most frequent polymorphism in genome, became the most favored DNA markers for linkage mapping and whole genome association studies in humans [12] and model organisms [13]. However, the application of SNPs in linkage mapping of aquacultured fish is just in its infancy [8].

Comparative mapping contributes largely to solve issues in the evolution of individual species, differences and similarities between various species and to characterize functions of genomes by providing a detailed analysis of conservation among orthologous intervals in different species [14]. This would not only help to reduce cost and increase efficiency in genetic research across species through offering the possibility to transfer genomic information available from model species to nonmodel organisms, but also aids whole genome sequence assembly as well as molecular breeding [15-17]. The availability of an increasing number of fish whole genome sequences (e.g., zebrafish, medaka, fugu, stickleback and freshwater pufferfish) offer opportunities to accelerate fish genomic comparative studies, such as, transferring genomic information from model species to food fish. Genetic mapping for non-model species with sequence-based molecular markers could provide a suitable support for the knowledge of genome organization and evolutionary studies by comparative map analysis. It represents a key towards integrating known genome data from model species into identifying genes in the species of interest [15,18,19].

The grass carp (*Ctenopharyngodon idella*) is one of the largest members of the family Cyprinidae [20,21]. It is an herbivorous freshwater fish of great commercial value and is extensively cultivated in Eastern Asia for food. Cultured grass carp may grow up fast reaching up to 1 kg in the first year, growing approximately 2-3 kg/year in temperate areas and 4.5 kg/year in tropical areas [20]. Grass carp also has extensive ecological adaptability, of the 115 countries in which grass carp have been introduced, at least 58 (~50%) appear to have self-sustaining populations [22]. In addition, due to their ability to be cultured easily, hardiness and effective biological controls on wide variety of aquatic vegetation; this species has been extensively used for aquatic weed control purposes in rivers, fish ponds and reservoirs [21]. In recent years, commercial harvesting of grass carp has increased in many countries. According to the statistics of Food and Agriculture Organization of the United Nations [23], the global annual production of grass carp summed up to 4,010,281 metric tons in 2006 with a 30% increase for past decades and was listed as the 3^rd ^biggest contributor to the world's aquaculture production. The revenue generated by aquaculture of this species was over 4 billion US dollars in 2006 [23]. Therefore, the species represents one of the most promising species in world aquaculture [24]. However, research on grass carp genomics remained at slow pace. Although to date around 100 microsatellite sequences of grass carp are available in GenBank database, and some microsatellite markers have been applied in genetic diversity and comparative analysis of grass carp [25-27], no genetic map is available in grass carp.

The aim of this study is to construct a first generation genetic map of grass carp using microsatellite and SNP markers to supply a basis for genome-wide search of QTL for economically important traits and to conduct a comparative mapping analysis to provide new insights into the evolution of fish genomes and to aid future genome assembly.

## Results

### Markers

A total of 283 microsatellites and 24 SNP markers were informative in the two mapping families and could be used for linkage analysis. Four microsatellite markers (*CID131A/B*, *CID320A/B*, *CID532A/B*, *CID816A/B*) were scored as duplicates.

### Linkage analysis

**Figure 1:**
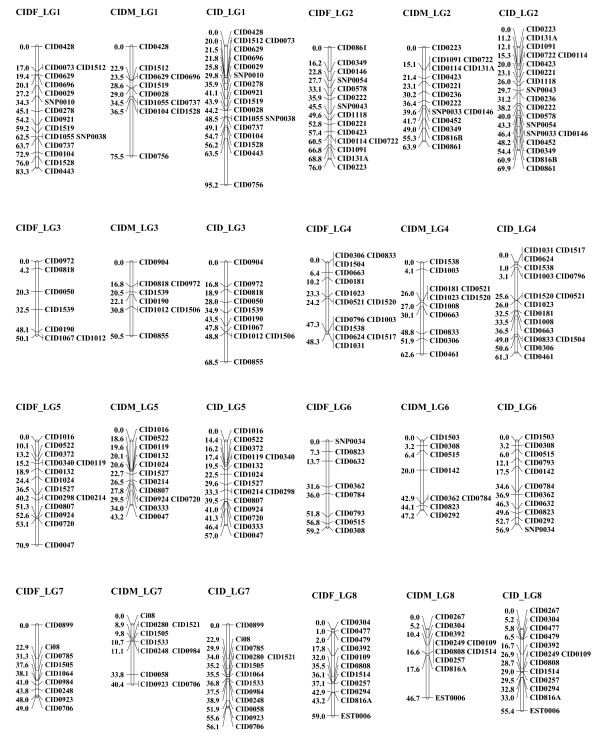
**Linkage groups 1 to 8 of the consensus linkage map of grass carp based on microsatellite and SNP markers**. The female linkage group (left) is named as "CIDF_LG" and 1-2 numbers; the male linkage group (middle) is named as "CIDM_LG" and 1-2 numbers; the sex-averaged linkage group (right) is named as "CID_LG" and 1-2 numbers. Estimates of map distances between markers are indicated in Kosambi centimorgans.

**Figure 2:**
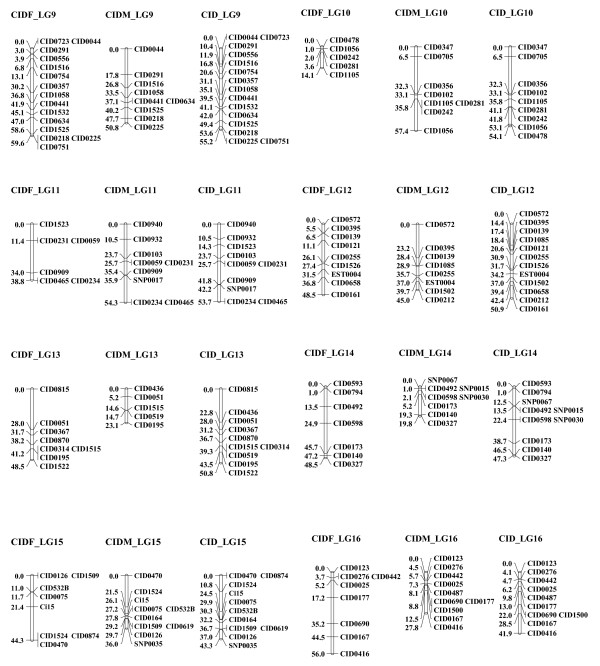
**Linkage groups 9 to 16 of the consensus linkage map of grass carp based on microsatellite and SNP markers**. The female linkage group (left) is named as "CIDF_LG" and 1-2 numbers; the male linkage group (middle) is named as "CIDM_LG" and 1-2 numbers; the sex-averaged linkage group (right) is named as "CID_LG" and 1-2 numbers. Estimates of map distances between markers are indicated in Kosambi centimorgans.

**Figure 3:**
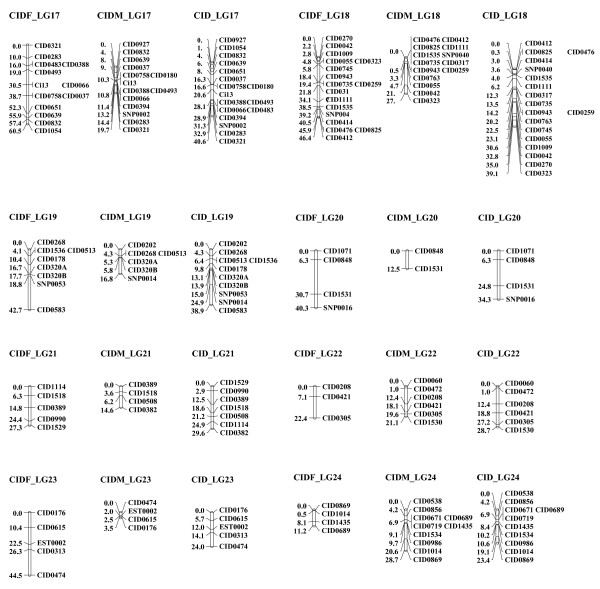
**Linkage groups 17 to 24 of the consensus linkage map of grass carp based on microsatellite and SNP markers**. The female linkage group (left) is named as "CIDF_LG" and 1-2 numbers; the male linkage group (middle) is named as "CIDM_LG" and 1-2 numbers; the sex-averaged linkage group (right) is named as "CID_LG" and 1-2 numbers. Estimates of map distances between markers are indicated in Kosambi centimorgans.

Two grass carp families containing 96 progenies in each were scored for 307 informative loci. A total of 279 markers (263 microsatellites and 16 SNPs) were mapped to 24 linkage groups (Additional file 1: Table S1) whereas 28 remained (20 microsatellites and 8 SNPs) unmapped. There is no significant deviation of Mendelian segregation for mapped markers after Bonferroni correction. Using linkage analysis and LOD ≥ 3 threshold, for male segregation data, 212 loci were finally assembled into 24 linkage groups (LGs) in both families, and for female data, 219 loci were assembled into 24 linkage groups in both families (Figures 1, 2 and 3 and Table 1). Among them, 152 loci were located in both maps which could be used to identify homologous pairs of linkage groups in both sexes. The merged sex-specific maps in both families showed different length. The map length for female and male was 1149.4 cM and 888.8 cM respectively. The average spacing between loci in male map (4.2 ± 1.7 cM) was lower than that in the female map (5.2 ± 1.8 cM), indicating that sex-specific differences in recombination rates existed in grass carp. Comparison of recombination differences between the parents of two mapping families revealed that the average recombination ratio (female: male) across all pairwise comparisons was 2.03 [N = 287 comparisons; G-test value (1 d.f) = 1132.2] for family 1 and 2.00 [N = 307 comparisons; G-test value (1 d.f) = 1226.9] for family 2. Therefore, a significantly higher recombination rate was evident in the female map but no significant family-specific difference in overall recombination ratio for both sexes was detected [Female (family1)/Female (family 2) = 1.02, N = 243 comparisons, G-test value (1 d.f) = 0.91; Male (family1)/Male (family2) = 1.01, N = 281 comparisons, G-test value (1 d.f) = 0.29]. The distributions of recombination ratio between both parents in two mapping families were given in Additional file 2: Figure S1.

**Table 1:** Number of markers and genetic length of sex-specific linkage groups of grass carp

*LG*	*Female*			*Male*		
	**No. of loci**	**Length (cM)**	**cM/marker**	**No. of loci**	**Length (cM)**	**cM/marker**

1	16	83.3	5.2	11	75.5	6.7
2	15	76.0	5.1	15	63.9	4.3
3	7	50.1	7.2	8	50.5	6.3
4	14	48.3	3.5	11	62.6	5.7
5	14	70.9	5.1	12	43.2	3.6
6	8	59.2	7.4	8	47.2	5.9
7	9	49.0	5.4	10	40.4	4.0
8	11	59.0	5.4	10	46.7	4.7
9	15	59.6	4.0	9	50.8	5.6
10	5	14.1	2.8	8	57.4	7.2
11	6	38.8	6.5	9	54.3	6.0
12	9	48.5	5.4	8	45.0	5.6
13	8	48.5	6.1	5	23.1	4.6
14	7	48.5	6.9	8	19.8	2.5
15	8	44.3	5.5	10	36.0	3.6
16	8	56.0	7.0	10	27.8	2.8
17	13	60.5	4.7	14	19.7	1.4
18	17	46.4	2.7	14	27.7	2.0
19	8	42.7	5.3	6	16.8	2.8
20	4	40.3	10.1	2	12.5	6.3
21	5	27.3	5.5	4	14.6	3.7
22	3	22.4	7.5	6	21.1	3.5
23	5	44.5	8.9	4	3.5	0.9
24	4	11.2	2.8	10	28.7	2.9
Total	219	1149.4	-	212	888.8	-
Average (± SD)	9.1 (± 4.2)	47.9 (± 17.4)	5.2 (± 1.8)	8.8 (± 3.3)	37.0 (± 18.9)	4.2 (± 1.7)

**Figure 4:**
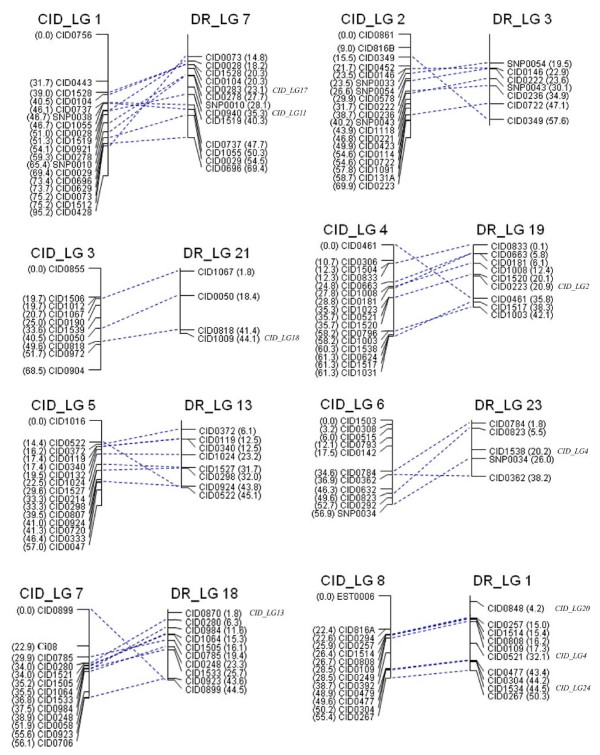
**Sex-averaged map (LG1 to 8) of grass carp (CID_LG) and synteny relationship to the assembled zebrafish genome sequence (DR_LG)**. Each vertical line represents individual grass carp linkage group (left) or zebrafish chromosome (right). Marker names and genetic distances (cM) of each linkage group were shown on left side, and the marker names and their physical distances (Mb) for zebrafish chromosome were given on right side. For the marker on the zebrafish chromosome without assignment to the current linkage group, its targeted LG on the grass carp linkage map was shown in italics on the right side of zebrafish chromosome.

**Figure 5:**
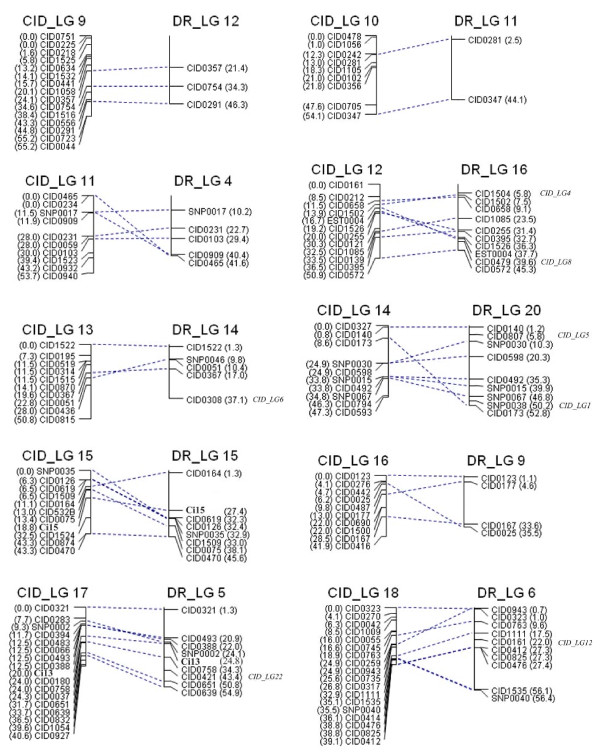
**Sex-averaged map (LG9 to 18) of grass carp (CID_LG) and synteny relationship to the assembled zebrafish genome sequence (DR_LG)**. Each vertical line represents individual grass carp linkage group (left) or zebrafish chromosome (right). Marker names and genetic distances (cM) of each linkage group were shown on left side, and the marker names and their physical distances (Mb) for zebrafish chromosome were given on right side. For the marker on the zebrafish chromosome without assignment to the current linkage group, its targeted LG on the grass carp linkage map was shown in italics on the right side of zebrafish chromosome.

**Figure 6:**
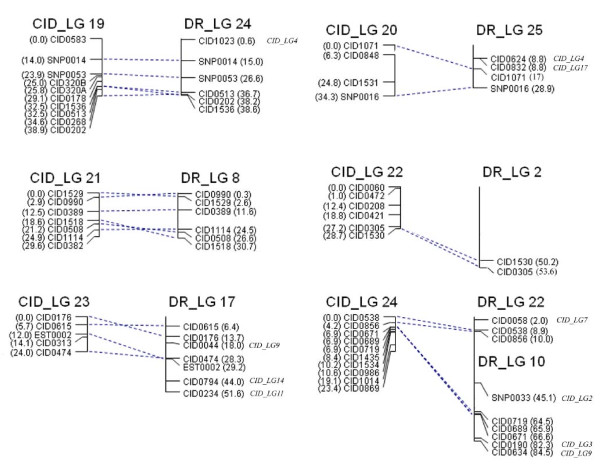
**Sex-averaged map (LG19 to 24) of grass carp (CID_LG) and synteny relationship to the assembled zebrafish genome sequence (DR_LG)**. Each vertical line represents individual grass carp linkage group (left) or zebrafish chromosome (right). Marker names and genetic distances (cM) of each linkage group were shown on left side, and the marker names and their physical distances (Mb) for zebrafish chromosome were given on right side. For the marker on the zebrafish chromosome without assignment to the current linkage group, its targeted LG on the grass carp linkage map was shown in italics on the right side of zebrafish chromosome.

The resulting sex-averaged map composed of 24 linkage groups (Figures 1, 2 and 3 and Figures 4, 5 and 6) contained 279 loci. Six (*CID131A*, *CID320A*, *CID320B*, *CID532B, CID816A *and *CID816B*) of the duplicate loci were assigned to 4 linkage groups (CID_LG2, 8, 15 and19) and the remaining two were unmapped (*CID131B *and *CID532A*). The marker order in the sex-averaged map was the same as in the sex-specific maps. The map spanned 1176.1 cM with individual linkage groups ranging from 23.4 to 95.2 cM (mean 49.0 ± 16.2, Table 2). The average resolution of the map was 4.2 ± 1.6 cM ranging from 2.2 cM (CID_LG18) to 8.6 cM (CID_LG20) for individual linkage groups. The average number of loci per linkage group was 11.6 ± 4.1 with a range varying from 4 (CID_LG20) to 19 loci (CID_LG2) per linkage group. Only 2 (0.9%) out of the 221 intervals were larger than 25 cM. Overall statistics including number of markers, linkage length and map length for the sex-specific and sex-averaged maps are given in Table 1 and 2.

**Table 2:** Summary of the sex-averaged linkage map of grass carp and the homologous zebrafish chromosomes that share at least two markers in common

*Grass carp linkage map*				*Zebra fish genome map*				
**LG**	**Length of LG (cM)**	**No. of loci**	**ALD (cM)**	**Homologous chromosome**^**1**^	**No. of syntenic loci**^**2**^	**Length of syntenic region (Mb)**	**Syntenic ALD (Mb)**	**R**

1	95.2	18	5.3	7	11	54.6	5.0	78%
2	69.9	19	3.7	3	7	38.1	5.4	60%
3	68.5	10	6.9	21	3	39.6	13.2	86%
4	61.3	16	3.8	19	8	42.0	5.3	91%
5	57.0	15	3.8	13	8	39.0	4.9	72%
6	56.9	11	5.2	23	4	36.4	9.1	79%
7	56.1	13	4.3	18	9	38.2	4.2	78%
8	55.4	13	4.3	1	7	35.3	5.0	63%
9	55.2	15	3.7	12	3	24.9	8.3	52%
10	54.1	9	6.0	11	2	41.6	20.8	92%
11	53.7	10	5.4	4	5	31.4	6.3	73%
12	50.9	12	4.2	16	8	37.8	4.7	71%
13	50.8	10	5.1	14	3	15.7	5.2	28%
14	47.3	10	4.7	20	7	51.6	7.4	91%
15	43.3	11	3.9	15	8	44.3	5.5	94%
16	41.9	10	4.2	9	4	34.4	8.6	67%
17	40.6	17	2.4	5	8	53.6	6.7	77%
18	39.1	18	2.2	6	9	55.7	6.2	94%
19	38.9	10	3.9	24	5	23.6	4.7	59%
20	34.3	4	8.6	25	2	11.9	6.0	36%
21	29.6	7	4.2	8	6	30.4	5.1	54%
22	28.7	6	7.8	2	2	3.4	1.7	6%
23	24.0	5	4.8	17	4	22.8	5.7	44%
24	23.4	10	2.3	10	3	2.1	0.7	5%
24	23.4	10	2.3	22	2	1.1	0.6	3%
Total	1176.1	279	--	--	138	809.5	--	63%

Average (± SD)	49.0 (± 16.2)	11.6 (± 4.1)	4.2 (± 1.6)	--	5.5 (± 2.7)	32.4 (± 14.6)	6.3 (± 4.0)	--

ALD, average inter-locus distance; '1' the homologous zebrafish chromosome that share at least two markers in common with a given grass carp linkage group; '2', the number of syntenic loci in one pair of grass carp LG and homologous zebrafish chromosome; R, the ratio of syntenic region to displayed region of reference chromosome (Mb) in NIH genetic sequence database http://blast.ncbi.nlm.nih.gov/Blast.cgi; Length of syntenic region (Mb) was calculated based on the distance between the longest syntenic pair of loci in each homologous zebrafish chromosome.

### Comparative mapping

The sequences of 263 mapped microsatellites and 16 SNPs in ESTs and gene loci were used for similarity searches against genome sequences of four model fish species. Our results showed that 10.1% (medaka sequences: 28 hits) to 59.2% (zebrafish: 164 hits) of the blasted sequences were significantly conserved between grass carp and model fish species (e < 10^-7^; Table 3). Between grass carp and fugu/medaka, no microsatellites with more than 5 or more repeat motif were conserved, whereas between grass carp and zebrafish, 43.9% of microsatellite loci were conserved. When compared to microsatellite data, much higher conservation (ranging from 43.8% for medaka to 100% for zebrafish) of homologous ESTs and gene loci between grass carp and model species was detected. Of the total 176 loci with significant hits (e < 10^-7^), 42 loci (23.9%) matched at least 2 model species and 13 (7.4%) matched all the 4 model species. By syntenic detection, some blocks with very high conservation were also found. For example, a syntenic block defined by markers *SNP0010 *and *CID0278 *on CID_LG1 was conserved among at least 3 species. The detailed comparative mapping results were presented in Additional files 3-4 Tables S2 and S3.

**Table 3:** Number of the conserved orthologous markers between grass carp and four model fish species

**Type**^**a**^	Zebrafish	Tetraodon	Fugu	Medaka
1	126	3	3	2
2	12	8	2	2
3	13	12	15	6
4	13	13	13	13
Total (%)^b^	164 (59.2%)	36 (13.0%)	33 (11.9%)	28 (10.1%)

'^a^', shows the conservation of orthologs, e.g., type 1 indicates unique orthologs identified for each model species, type 2 shows orthologs identified only in two model species (the given model species and another model species), type 3 shows orthologs identified in three model species (the given model species and other two model species), etc; ^'b'^, ratio of all orthologous markers to the total informative markers used for similarity searches.

The grass carp genetic map was utilized in the establishment of gene orthology relationships based on conserved syntenies among species. The zebrafish genome with 25 chromosomes was fairly characterized. One hundred and sixty-four (148 microsatellites and 16 SNPs) markers were common in grass carp linkage map and zebrafish genome map. The marker density in the comparative map ranged from 2 (CID_LG10) to 13 (CID_LG1 and CID_LG4) markers with an average of 6.8 ± 3.0 per LG (Table 4). These allow us to evaluate large-scale synteny patterns between the two species.

**Table 4:** Macrosyteny relationship between grass carp and zebrafish genome

	C1	C2	C3	C4	C5	C6	C7	C8	C9	C10	C11	C12	C13	C14	C15	C16	C17	C18	C19	C20	C21	C22	C23	C24	Total
D1				1				7												1				1	10
D2																						2			2
D3		7																							7
D4											5														5
D5																	8					1			9
D6												1						9							10
D7	11										1						1								13
D8																					6				6
D9																4									4
D10		1	1						1															3	6
D11										2															2
D12									3																3
D13					8																				8
D14						1							3												4
D15															8										8
D16				1				1				8													10
D17									1		1			1									4		7
D18							9						1												10
D19		1		8																					9
D20	1				1									7											9
D21			3															1							4
D22							1																	2	3
D23				1		4																			5
D24				1															5						6
D25				1													1			2					4

Total	12	9	4	13	9	5	10	8	5	2	7	9	4	8	8	4	10	10	5	3	6	3	4	6	164
Chr ^a^	2	3	2	6	2	2	2	2	3	1	3	2	2	2	1	1	3	2	1	2	1	2	1	3	--

"D1-25" refer to the 25 zebrafish chromosomes and "C1-24" refer to the 24 grass carp linkage groups, respectively; '^a^', the zebrafish chromosome number that shows synteny to a given grass carp linkage group.

All 24 grass carp LGs were clearly associated with zebrafish chromosomes (Figures 4, 5 and 6 and Table 4). In most cases, the grass carp LG showed extensive synteny towards a particular zebrafish chromosome, sharing two or more loci with zebrafish chromosome. The average number of syntenic loci in one pair of grass carp LG and homologous zebrafish chromosome was 5.5 ± 2.7 with a range varying from 2 (CID_LG10-DR_LG11, CID_LG20-DR_LG25, CID_LG22-DR_LG2 and CID_LG24-DR_LG22) to 11 (CID_LG1-DR_LG7) per chromosome (Table 2). In total, 6 of the 24 grass carp LGs (CID_LG10-DR_LG11, CID_LG15-DR_LG15, CID_LG16-DR_LG9, CID_LG19-DR_LG24, CID_LG21-DR_LG8, CID_LG23-DR_LG17) mapped each to a single zebrafish chromosome, 12 yielded hits with 2 chromosomes, 5 with 3 chromosomes and one (CID_LG4) mapped to 6 chromosomes (Figures 4, 5 and 6 and Table 4).

Each zebrafish chromosome was mainly syntenic to one grass carp LG and shared ≥50% orthologous markers of the chromosome in common (Figures 4, 5 and 6). The zebrafish genome map covered by these syntenic markers summed up to 809.5 Mb with a range of 1.1 Mb (DR_LG22) to 55.7 Mb (DR_LG6) per chromosome, accounting for 63% of the displayed regions of zebrafish reference genome (Table 2). A large amount of syntenic blocks could be defined by two or more syntenic loci in this study. This showed substantial macrosynteny relationship existed between the two species.

The extensive colinearity was very evident for some syntenic chromosomes/linkage pairs e.g. CID_LG3-DR_LG21, CID_LG5-DR_LG13, CID_LG8-DR_LG1, CID_LG9-DR_LG12, CID_LG10-DR_LG11 but partial collinearity was more frequently observed for other chromosomes/linkage pairs, indicating large-scale chromosomal rearrangements were rare but small inversions and translocations were prevailing. However, there were few exceptions e.g. both of the zebrafish chromosomes, DR_LG10 and DR_LG22, were mainly syntenic to one grass carp linkage group, CID_LG24 and shared ≥50% orthologous markers of the chromosome. In addition, 16 SNP markers mapped to 10 grass carp LGs were assigned to 10 chromosomes of the zebrafish genome map of which, 14 (87.5%) were found in syntenic regions (defined with ≥50% orthologous markers). However, for the 263 mapped microsatellite markers, only 47.5% were found in syntenic regions. This data indicates a strong conservation of functional genes in the two species.

## Discussion

### DNA markers and genetic maps

Genetic maps are essential tools for fish genomic studies [28]. Development of a large number of sequence-based genetic markers including microsatellites and SNPs is necessary for mapping QTLs for traits of interest and carrying out marker-assisted selection (MAS). Using 307 informative DNA makers, we have constructed a first generation linkage map of grass carp including 263 microsatellites and 16 SNPs. Due to difficulty in scoring, 28 markers were not assigned to the linkage map. Two hundred and seventy-nine loci were arranged into 24 linkage groups. Grass carp is a diploid fish with 2n = 48 chromosomes [29,30], therefore the linkage group number is corresponding to the haploid chromosome number of grass carp. According to our best knowledge, this map is the first linkage map of a food fish species in the family Cyprinidae exclusively based on co-dominant markers (i.e. microsatellites and SNPs).

The genome size of grass carp estimated with a haploid C-value of 1-1.09 pg [31] and the conversion formulas (1 pg = 978 Mb) [32] was 978-1066 Mb. The length of the grass carp linkage map was 1176.1 cM. Therefore, the ratio between physical and linkage distance was 832 - 906 kb/cM. This figure is similar to some other fishes, such as rainbow trout [16], but higher than that of turbot (530 kb/cM)[19] and tiger pufferfish (420 kb/cM) [33]. The length of the grass carp map is far less than the linkage map of the common carp (*Cyprinus carpio*; 4111 cM)[34], but similar to the length of silver carp (*Hypophthalmichthys molitrix*; 1150 cM) and bighead carp (*Aristichthys nobilis*; 1209 cM) genetic maps [35].

Compared to density of first-generation linkage maps of other species, such as the turbot (6.5 cM) [19], tiger pufferfish (7.1 cM) [33], Barramundi (4.7-6.2 cM) [9] and olive flounder (4.7 cM) [36], the average resolution of our map was slightly better with an average distance of 4.2 (± 1.6) cM between loci. Therefore, such a map would be dense enough for the initial mapping of interesting agriculture traits and facilitate future comparative genomic analyses for understanding genome evolution. Chinese scientists are planning to sequence the complete genome of grass carp. Therefore, this linkage map could be complementary to a genome sequencing project.

### Sex recombination ratio

It is important to know the relative rates of recombination for both sexes of any species [37]. Significant differences in sex recombination ratios have been reported for many fishes such as rainbow trout (F:M, 1.68:1 and 3.25:1) [16,38], zebrafish (2.74:1.0) [37], Atlantic halibut (1.89-2.53:1) [39], European sea bass, *Dicentrarchus labrax *(1.5:1) [40], turbot (1.6:1) [19], Barramundi, *Lates calcarifer *(2.06:1) [9]. Family-specific recombination rates were also demonstrated in some salmon species [41]. These studies have shown that reduced male recombination rates might exist extensively in teleost fishes although it varied greatly among chromosomes and sub-chromosomal regions. The female:male recombination rates (~ 2:1) for the parents of two families in this study are similar to the ratios that were reported previously. However, we observed some exceptions for some marker intervals with higher recombination rates in male, which have also been reported in some fishes, such as zebrafish, trout and Barramundi [9,37,38]. Previous studies suggested many factors such as pericentromeric suppression, CpG islands, GC content, polyA/polyT content, simple repeats, LINE, SINE elements and other sequence features would influence recombination rate, however the molecular mechanisms responsible for the difference between the two sexes are still not well understood [3,37].

### Comparative mapping

Both grass carp and zebrafish belong to the family Cyprinidae with the divergence time of 63 ± 2 million years (Mya) [26] while the time of divergence between zebrafish and Takifugu was 290 ± 6 Mya [42]. The similarity searches against 4 model fish species reference genomes showed that 59.2% grass carp sequences used were considered to be significantly similar to zebrafish genome sequences. However, much lower significant hits ranging from 10.1% to 13.0% in Tetraodon, fugu and medaka was found. The result is in agreement with phylogenetic data [42], showing zebrafish was more closely related to grass carp than other model species.

Karyotype evolution in teleosts occurred mainly by inversion and not by fusion or fission of chromosomes [33,43]. Since grass carp (2n = 48) and zebrafish (2n = 50) have different karyotyes and genome sizes (C-value of 1-1.09 pg for grass carp and 1.68-2.28 pg for zebrafish) [31], the strict pairwise synteny of chromosome pairs between grass carp and zebrafish was not expected. Our study demonstrated extensive colinearity for some syntenic chromosome/linkage pairs. For example, each zebrafish chromosome was mainly syntenic to one grass carp LG and shared ≥50% othologous markers of the chromosome in common. This study also indicated that substantial macrosynteny relationship, inversions and translocations existed between grass carp and zebrafish. Therefore, our results provide further support to the studies of karyotype evolution in teleosts by Jaillon *et al*. [43] and Kai *et al. *[33]. However, due to the precision in marker order on the current grass carp linkage map was not high enough for some markers, and reference sequences were not free of assembly error [44], it was possible that some of the disagreements in marker order between grass carp and zebrafish were not accurate, which can be confirmed by further cytogenetic studies, such as FISH experiments.

Comparative genomics had shown the existence of several blocks of synteny between zebrafish and human gene maps [45]. Our study showed a strong conservation of gene order between grass carp and zebrafish and other model species. For example, 87.5% of the gene-based SNP markers and 47.5% of the flank sequence-based microsatellite markers were found in syntenic regions of grass carp to zebrafish; and 23.9% of the loci with significant hits matched at least 2 model species and 7.4% matched 4 model species. However, the conservation was different for most of the compared chromosomes between grass carp and zebrafish. This could be reflected by differentiated homologous zebrafish chromosome number that shows synteny to different grass carp linkage group (ranging from 1 to 6 chromosomes for different grass carp LG). Some studies had reported that chromosomes might be functionally partitioned [46] and large-scale genomic rearrangements were nonrandom with nucleotide variation [47]. Bourque *et al*. [48] also suggested that the total number of microrearrangements was much higher for anonymous sequence-based data than for gene-based data, as many of the microrearrangements in sequence-based synteny blocks lay outside exons. These studies demonstrated that conservation of synteny might reflect important functional relationships at chromosomal levels.

Synteny among species or genera might also aid initial QTL experiments with candidate gene approaches [49]. In this study, 16 SNPs derived from functional genes or ESTs related to complex genetic basis for interesting quantitative traits in productions such as muscle myosin heavy chain (MHY; SNP0038), TNF-related apoptosis inducing ligand (TRAIL; SNP0053) and c-Fos (c-fos; SNP0067) were mapped on to the linkage map at high confidence level. Most of them were also assigned to the conserved synteny regions of grass carp linkage map to zebrafish genome. The orthologous markers closely linked to these genes on conserved chromosomes of related model fish species might be considered to identify candidate genes and carry out MAS in further breeding strategies of the grass carp.

## Conclusions

We constructed a first generation linkage map containing 279 DNA markers (263 microsatellites and 16 SNPs in ESTs). The marker density was 4.20 cM/locus. Recombination rate was higher in females than in males. This linkage map will provide important base for mapping QTL affecting economically important traits and could be complementary to grass carp genome sequencing project. Comparative mapping between the grass carp and other model organisms revealed that substantial macrosynteny relationship and extensive colinearity existed between grass carp and zebrafish. Comparative mapping will facilitate understanding genome evolution. In near future the identification of QTL associated traits, such as related to disease and parasite resistance and growth rate, would be conducted with the markers on the map. Our study also showed strong syntenic relationships and a number of conserved colineality existed between grass carp and zebrafish genome. Therefore, gene prediction directly from zebrafish to grass carp was possible. Further studies focusing on the transferring of genomic information available from zebrafish to the selection of grass carp had been put forward. A high density map with gene-based markers was also planned, which would provide a detailed analysis of conservation among orthologous intervals in related species and a basis for location of genes and MAS of grass carp or other Cyprinidae.

## Methods

### Mapping families and DNA isolation

Prior to construction of reference mapping families, 150 candidate grass carp parents (75 males and 75 females) derived from wild populations in Yangtze and Pearl river systems were genotyped with 6 microsatellite markers (*Ci02, Ci08, Ci09, Ci11*, *Ci12 *and *Ci15*) as described previously [50] and 3 microsatellite markers (*CID0025, CID0029 *and *CID0037*) developed in this study. Genetic relatedness was calculated on the basis of microsatellite genotypes [51]. Two pairs of grass carps with the most genetic difference were selected for artificial insemination to produce F1 offspring. From each pair, Two millions of fertilized eggs were obtained and 96 progenies at the age of one month were randomly collected and stored in 75% ethanol for subsequent linkage analyses. Parental fin clips of 192 offspring from two families were sampled for DNA extraction with the method described as in Yue and Orban [52].

### Development and genotyping of DNA markers

Two microsatellite-enriched partial genomic libraries (CA and GA repeats) were constructed using DNA from one adult grass carp according to method described by Yue *et al *[53] with some minor modifications [50]. From each library, 2000 clones were sequenced in both directions with M13 and M13 reverse primers and BigDye chemicals on an ABI 3730×l Genetic Analyzer (Applied Biosystems). The microsatellite loci containing seven or more repeat units and enough flanking regions were identified for primer design using the program PrimerSelect (DNASTAR, Wilmington, DE). In addition, 17 microsatellites from a previous publication [50] were selected. Microsatellites were named as "CID" followed by 4 numbers while duplicate markers were named as "CID" and 3 numbers plus an "A" or "B". In addition, six EST-derived microsatellite loci were chosen by scanning the grass carp EST database in GenBank and their primers flanking repeats were developed. Three of them were mapped and given names as "EST" followed by 4 numbers.

To optimize PCR for each microsatellite, PCR reaction was performed in 25 μL volume containing 10 ng genomic DNA, 1×PCR buffer, 100 μmol of each dNTPs, 0.2 μmol forward primer, 0.2 μmol reverse primer and 1 U of *Taq *DNA polymerase (FINNZYMES, Espoo, Finland) on a thermal cycler (MJ Research, CA, USA) with the following cycling profile: one denaturation step for 2 min at 95°C was followed by 35 cycles of 30 sec at 94°C, 30 sec at 50°C, 55°C or 60°C and 45 sec at 72°C. The final step was a prolonged extension of 5 min at 72°C. PCR products were resolved on 2% agarose gel and visualized by ethidium bromide staining. Five hundred and ten primer that pairs amplified clear and strong bands were selected for labeling with fluorescent dyes. For each microsatellite, one primer was labeled either FAM or HEX (1^st ^Base, Singapore). These microsatellite markers were selected for genotyping parents. Finally 263 microsatellite markers, being informative among four parents, were selected for genotyping the two mapping families comprising 192 individuals. Microsatellites were amplified on grass carp genomic DNA with fluorescent primers under the same conditions used for amplification with unlabeled primers. PCR products were resolved on ABI3730×l Genetic Analyzer (Applied Biosystems, CA, USA) and were sized relative to an internal size standard (GeneScan-500 ROX) using GeneMapper 3.5 software package (Applied Biosystems, CA, USA) as described previously [54].

SNPs in ESTs were detected by PCR amplification of DNA of four parents and sequencing of PCR products. Briefly, 63 ESTs from grass carp were downloaded from GenBank and were aligned with genomic sequence data from zebrafish on GenBank. Primer sites in conserved exon regions were identified and a total of 63 primer pairs allowing PCR amplification of an intron-spanning fragment were developed. The primer sets were given names as "SNP" followed by 4 numbers in the genetic map. Fifty-five primer pairs amplified intron-spanning fragments which were sequenced as described above to detect SNP in the four parents. SNPs were detected in 16 ESTs. The genotyping for the 16 polymorphic SNP markers were performed based on ABI PRISM^® ^snapshot™ Multiplex Kit (Applied Biosystems, CA, USA) according to the manufacturer's protocol. Two to six SNP primers were combined in multiplex PCR reaction and the resultant snapshot products were resolved on ABI 3730×l Genetic Analyzer (Applied Biosystems) after post-extension treatment with Shrimp Alkaline Phosphatase (Promega, Madison, WI). Genotyping of PCR products were carried out against the internal size standard GeneScan-120 LIZ using GeneMapper 3.5 software package (Applied Biosystems, CA, USA).

Details of polymorphic markers in parents used to construct the grass carp genetic map were summarized in Additional file 1: Table S1 and the sequences were submitted to GenBank with accession nos FJ883175-FJ883463.

### Map construction

The sex-specific linkage maps for two mapping families were constructed independently using the program LINKMFEX (ver. 1.5) [55]. Genotype data for both families were checked for inconsistencies with Mendelian inheritance and manually corrected for error. Those markers with LOD ≥ 3 for segregation data were assigned to the same linkage group for both mapping families respectively using a two-point analysis. Map distances were estimated for each best likely order linkage group using the Kosambi function with the module MAPDIS. Integration of the sex-specific linkage maps and construction of a sex-averaged map for two mapping families was performed using the module MERGE. Finally, the sex-averaged linkage groups were numbered based on their group length (from large to small, namely, CID_LG1 to CID_LG24). The maps were visualized using MapChart software (ver. 2.1) [56]. For assessing the differences in recombination rate between sexes, comparisons of recombination differences between both parents in two mapping families were performed by analyzing all pairwise marker combinations using a two-way contingency G-test as implemented in the module RECOMDIF of the program LINKMFEX.

### Comparative mapping

The flanking sequences of polymorphic microsatellite loci and SNPs in ESTs and gene loci among parents were used for similarity searches against the zebrafish genomic sequences via the NIH genetic sequence database and against medaka, fugu and tetraodon genomic sequences via the Ensembl Genome Browser under default settings. Hits with e < 10^-7 ^were considered as significant. In cases where the searches hit two or more scaffolds or loci with less than 100 fold difference in the E-value, the genes were considered to be duplicated within a genome, in a conservative way, no orthology were assigned. The comparative map was drawn using MapDisto software (ver. 1.7) [57].

## Abbreviations

LG: linkage group; AFLP: amplified fragment length polymorphism; RAPD: random amplification of polymorphic DNA; SNP: single-nucleotide polymorphism; QTL: quantitative trait loci; EST: expressed sequence tag; MAS: marker-assisted selection; FISH: fluorescent in situ hybridization.

## Authors' contributions

GHY and JLL initiated the study. JHX, FL, ZYZ, JJF, JBF and GHY carried out the experimental and molecular work. JHX conducted the data analysis and drafted the bulk of the manuscript. All authors read and approved the final manuscript.

## Supplementary Material

Additional file 1 **Table S1**. Primer sequences of markers used in the construction of a linkage map of grass carp. Click here for file

Additional file 2 **Figure S1**. Distribution of recombination ratio between both parents in two grass carp mapping families. Click here for file

Additional file 3 **Table S2**. Putative othologous locus between the grass carp and four model fish species genomes. Click here for file

Additional file 4 **Table S3**. Putative othologous locus between the grass carp and zebrafish genomes. Click here for file
