# Supplementary material for: A consensus linkage map of the grass carp (Ctenopharyngodon idella) based on microsatellites and SNPs
Source: BMC Genomics. 2010 Feb 24;11:135. doi: 10.1186/1471-2164-11-135 (PMC2838847; doi:10.1186/1471-2164-11-135)
Supplement: Additional file 2 — Figure S1. Distribution of recombination ratio between both parents in two grass carp mapping families. [file 1471-2164-11-135-S2.DOC]

**Fig. S1 Distribution of recombination ratio between both parents in two grass carp mapping families**

Fig.S1a shows the recombination ratio (Female: Male) across all pairwise comparisons; Fig.S1b shows the family-specific differences [(Female (family1) / Female (family 2); Male (family1) / Male (family2)] in overall recombination ratio for both sexes. The recombination ratio is taken as the ratio of the recombination distance in first parent to that obtained from the second parent. Where a zero recombination rate was observed in the second comparison mapping parent, the recombination ratio was designated as ‘50’ for plotting. Analysis ignored duplicated marker designations between both mapping parents. Comparisons of recombination differences between both parents in two mapping families were performed by analyzing all pairwise marker combinations using a two-way contingency G-test as implemented in the module RECOMDIF of the program LINKMFEX.
