# Supplementary material for: A consensus linkage map of the grass carp (Ctenopharyngodon idella) based on microsatellites and SNPs
Source: BMC Genomics. 2010 Feb 24;11:135. doi: 10.1186/1471-2164-11-135 (PMC2838847; doi:10.1186/1471-2164-11-135)
Supplement: Additional file 3 — Table S2. Putative othologous locus between the grass carp and four model fish species genomes. [file 1471-2164-11-135-S3.DOC]

Table S2 Putative othologous loci between genomes of the grass carp and four model fish species

| *Marker name* | *LG (cM) in Grass carp* | *Chromosome (Mb) in Zebrafish* | *Chromosome*  *(Mb) in Tetraodon* | *Position in Fugu* | *Position in Medaka* |
| --- | --- | --- | --- | --- | --- |
| SNP0043 | 1(27.7) | 3(30.1) | Un_random (4.6) | scaffold253 | - |
| SNP0033 | 1(39.9) | 10(2.1) | Un_random (6.5) | scaffold209 | - |
| SNP0010 | 2(16.9) | 7(28.1) | 5(1.3) | scaffold28 | scaffold67 |
| CID0278 | 2(17.5) | 7(43.4) | - | scaffold28 | scaffold67 |
| CID1512 | 2(22.4) | - | - | - | scaffold6775 |
| CID0629 | 2(23.2) | - | - | - | scaffold6775 |
| CID0696 | 2(23.9) | 7(47.1) | 17(1.9) | scaffold6 |  |
| SNP0038 | 2(38.8) | 20(50.2) | Un_random (110.4) | scaffold139 | scaffold3716 |
| CID0102 | 4(14.9) | - | 13(1.3) | scaffold9731 | scaffold328_1 |
| CID0347 | 4(56.2) | 11(10.7) | 2(15.0) | - | - |
| CID0705 | 4(60.5) | - | - | scaffold57 | - |
| CID1539 | 5(35.6) | 21(14.6) | - | scaffold179 | scaffold191 |
| CID1503 | 6(0.0) | - | Un_random (21.3) | - | - |
| CID0515 | 6(3.9) | - | Un_random (70.8) | - | - |
| CID0308 | 6(6.0) | 14(20.9) | - | - | scaffold5289 |
| CID0823 | 6(38.5) | 23(0.1) | - | scaffold109 | - |
| SNP0034 | 6(51.2) | 23(26.0) | - | scaffold20 | scaffold209 |
| CID0291 | 7(36.4) | 12(45.6) | 1(1.1) | - | - |
| CID0044 | 7(38.3) | 17(18.0) | 2(0.7) | scafold 29 | scaffold19 |
| CID0754 | 7(40.4) | 12(16.2) | 2(0.8) | - | scaffold148 |
| CID0556 | 7(51.1) | - | 17(6.2) | - | - |
| SNP0017 | 8(16.0) | 4(10.2) | 13(10.5) | scaffold2 | scaffold30 |
| CID0058 | 9(0.0) | 22(14.8) | 1(16.2) | - | - |
| CID0923 | 9(18.0) | 18(0.7) | - | scaffold1 | scaffold5 |
| CID0706 | 9(18.6) | - | - | scaffold490 | - |
| CID0899 | 9(23.0) | 18(30.3) | 13(11.8) | scaffold418 | - |
| CID0195 | 10(42.0) | - | Un_random(78.6) | scaffold70 | scaffold1674 |
| CID0870 | 10(44.7) | 18(43.6) | 1(0.5) | scaffold203 | scaffold159 |
| CID0519 | 10(47.8) | 7(37.1) | Un_random(37.5) | scaffold2268 | scaffold214 |
| CID0833 | 12(1.0) | 19(4.5) | 3(0.2) | - | - |
| CID1504 | 12(3.0) | 16(5.8) | Un_random (47.8) | scaffold2087 | scaffold89 |
| CID0461 | 12(11.5) | 19(19.4) | - | scaffold12 | scaffold44 |
| CID1538 | 12(43.3) | - | 21_random(0.4) | scaffold69 | scaffold8 |
| CID0470 | 13(39.6) | 15(5.8) | Un_random(19.5) | scaffold188 | - |
| SNP0067 | 15(18.3) | 20(46.8) | 10(5.24) | scaffold193 | - |
| SNP0030 | 15(22.9) | 20(10.3) | Un_random(70.5) | - | - |
| CID0025 | 16(32.4) | 9(35.5) | 2(13.7) | - | - |
| CID0123 | 16(38.4) | 9(33.7) | - | scaffold36 | - |
| CID0321 | 17(38.3) | 5(45.1) | - | - | scaffold28 |
| CID0268 | 18(5.1) | - | - | scaffold80 | - |
| SNP0053 | 18(12.1) | 24(26.6) | Un_random(89.6) | - | - |
| SNP0014 | 18(19.8) | 24(15.0) | 13(12.9) | scaffold152 | scaffold22 |
| CID1531 | 19(26.0) | - | 13(6.6) | scaffold2 | scaffold54 |
| SNP0016 | 19(36.3) | 25(28.9) | Un_random(103.2) | scaffold91 | scaffold680 |
| EST0002 | 20(17.3) | 17(37.7) | 10(2.8) | scaffold52 | scaffold115 |
| EST0004 | 21(30.6) | 16(7.7) | Un_random(91.7) | scaffold61 | scaffold164 |
| CID0825 | 22(0.2) | 6(4.2) | 2(14.3) | - | scaffold450 |
| CID0412 | 22(0.4) | 6(22.9) | Un_random(112.0) | scaffold258 | scaffold6 |
| SNP0040 | 22(5.5) | 6(56.4) | 1(22.9) | scaffold2399 | scaffold23 |
| CID1529 | 24(20.7) | 8(2.6) | 12(11.9) | - | - |

The position in each model species genome of the putative othologous locus identified using BLASTn was provided. For zebrafish only 38 othologous loci that conserved between zebrafish and one or more model species were shown. All of the 164 orthologs that conserved between the grass carp and zebrafish genome (including the 126 unique orthologs identified for zebrafish) were provided in Table S3. “-“: no match.
